# Kinematic and Muscle Activation Differences Between High-Performance and Intermediate Tennis Players During the Forehand Drive

**DOI:** 10.3390/s26072244

**Published:** 2026-04-04

**Authors:** Bruno Pedro, Silvia Cabral, Filipa João, Andy Man Kit Lei, António P. Veloso

**Affiliations:** CIPER, Faculdade de Motricidade Humana, Universidade de Lisboa, Cruz-Quebrada-Dafundo, 1499-002 Lisbon, Portugal; bmpedro@fmh.ulisboa.pt (B.P.); scabral@fmh.ulisboa.pt (S.C.); filipajoao@fmh.ulisboa.pt (F.J.); mklei@edu.ulisboa.pt (A.M.K.L.)

**Keywords:** upper limb biomechanics, IMU joint kinematics, EMG amplitude, racket velocity

## Abstract

This study compared the kinematic and neuromuscular characteristics of the tennis forehand drive between high-performance (HP) and intermediate (INT) players. Eighteen right-handed male players (HP: *n* = 9; INT: *n* = 9) performed cross-court forehands while three-dimensional motion capture and surface electromyography (EMG) were recorded from the dominant upper limb and trunk. Kinematic and EMG data were time-normalized to the forward swing. One-dimensional statistical parametric mapping two-sample *t*-tests were used to compare joint angles, angular and linear velocities, and EMG amplitude waveforms between groups. Bonferroni-corrected significance levels were set at α = 0.0017 for kinematic variables and α = 0.0063 for EMG data. HP players exhibited greater racket linear velocity during the final part of the forward swing, accompanied by higher shoulder, elbow and wrist linear velocities, whereas hip linear velocity did not differ between groups. Joint angles were broadly similar, with SPM revealing only slightly greater early knee flexion in HP players. In contrast, HP players showed higher hip and knee angular velocities and greater wrist angular velocities in both flexion/extension and radial/ulnar deviation towards impact. EMG patterns were generally comparable, but HP players displayed higher biceps brachii activation in two significant clusters during the mid-to-late forward swing and greater triceps brachii activation in the late forward swing. No significant differences were observed for deltoid, pectoralis major, latissimus dorsi, flexor carpi radialis or extensor carpi radialis. These findings indicate that superior forehand performance in HP players is associated primarily with refined segmental coordination, greater lower-limb and distal segment velocities, and locally increased elbow muscle activation, rather than with widespread increases in upper-limb or trunk muscle activity.

## 1. Introduction

The tennis forehand is among the earliest and most fundamental strokes taught to novice players [1], and it remains one of the most frequently used and decisive techniques at the professional level [2]. Performing an effective forehand requires precise temporal and spatial coordination across multiple joints, particularly in the trunk and upper extremities. This coordination enables efficient force transfer along the kinetic chain and ultimately to the racket at the instant of ball contact [3,4]. While the external motion of the stroke appears smooth and continuous, it is underpinned by complex neuromuscular activity patterns that remain incompletely understood.

Kinematic studies have identified notable differences in forehand technique between players of different performance levels [2,4], and reported differences in joint angles and timing of peak angular velocities between elite and high-performance athletes. These biomechanical differences suggest that distinct neuromuscular strategies may underlie stroke performance across skill levels. However, most existing EMG studies have focused on movement mechanics or on muscle activation during task variation, such as topspin versus flat forehands [5], changes in racket mass [6], or fatigue effects [7], without directly comparing players of different competitive levels.

Other studies of the tennis forehand have highlighted key patterns of muscle activation demonstrating that the greatest EMG activity occurred during racket acceleration and impact, particularly in the pectoralis major, deltoid, biceps brachii, and the flexor pollicis brevis [8]. More recently, it was confirmed that the flexor carpi radialis (FCR) can reach activation levels near 100% of maximal voluntary contraction (MVC), and the extensor carpi radialis (ECR) up to 80%, during the forehand [9]. Furthermore, it was observed that INT players tended to exhibit higher FCR activity, whereas advanced players demonstrated greater ECR activation, suggesting possible differences in technical execution and muscle recruitment strategies [10].

Despite these findings, a clear gap remains in the literature: no study to date has systematically compared forehand EMG patterns between HP- and INT-level players. Understanding such differences is critical, as inefficient muscle recruitment may increase the risk of overuse injuries—particularly in the wrist and forearm, where ulnar-sided pathologies such as extensor carpi ulnaris tendinopathy are prevalent [11]. Additionally, knowledge of how muscle coordination varies with skill level may inform targeted coaching, conditioning, and rehabilitation protocols.

In the field of motion analysis, 3D stereophotogrammetry is currently recognized as the gold standard for kinematic evaluation during walking, as previous articles reported [12,13]. However, while optoelectronic systems offer extreme precision, their application is often restricted to controlled laboratory environments, which may limit the ecological validity of sports-specific tasks. In this context, IMUs represent an ideal solution due to their portability, cost-effectiveness, and satisfactory repeatability in dynamic conditions. In fact, the literature [14] reports that IMU-based systems provide a level of repeatability and accuracy that is highly suitable for dynamic motor tasks and technical evaluation in real-world settings in comparison with the gold standard instrumentation.

To address the lack of knowledge, this study aims to compare the kinematic and EMG amplitude in upper-limb and trunk muscles between HP and INT tennis players during the tennis forehand drive. It was hypothesized that the upper-limb and trunk muscle activity would differ between player levels. Also, it was hypothesized that the HP players present a higher racket velocity compared to the INT tennis players.

## 2. Materials and Methods

### 2.1. Participants

A convenience sample of eighteen male tennis players, nine right-handed HP (age: 19.7 ± 7.3 years; height: 175.9 ± 5.9 cm; mass: 66.1 ± 5.6 kg; body mass index (BMI): 21.4 ± 1.9 kg/m^2^) and eight right-handed and one left-handed INT (age: 17.0 ± 3.4 years; height: 172 ± 8.3 cm; mass: 59.8 ± 10.1 kg; BMI: 20.0 ± 2.2 kg/m^2^), was used. The HP players had been practicing regularly for 12.2 ± 9.6 years before the study and their average weekly training during the 6 months before testing was 8.7 ± 2.7 h/week, and the INT players had been practicing regularly for 5.21 ± 2.2 years before the study and their average weekly training during the 6 months before testing was 2.6 ± 0.8 h/week. All participants were injury-free. The HP tennis players competed in national competitions and held a national ranking, while the INT did not compete and had no national ranking.

### 2.2. Experimental Procedures

**Figure 1:**
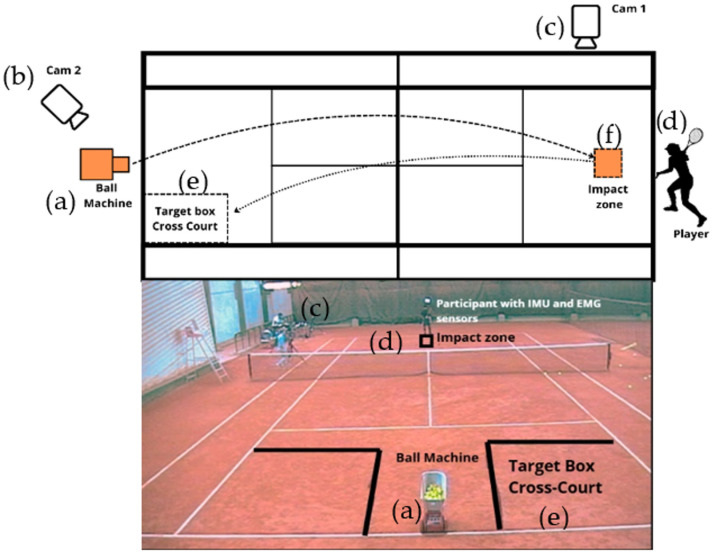
Set up environment: (**a**) ball machine; (**b**) panasonic digital video camera (cam 2); (**c**) qualisys Oqus 210c camera (cam 1); (**d**) participant with the Xsens IMU’s; (**e**) target box for the cross-court direction forehands; (**f**) impact zone.

Prior to data collection all participants provided written informed consent to participate in this study, which was approved by the Institution’s Ethics Committee (21/2018), and observed a demonstration of the experimental procedure. They had 15 min for an individual warm-up, and they could hit as many strokes as they wanted to become familiar with the test environment [4]. Participants used their own tennis rackets to ensure they felt as comfortable as possible during the test. Each participant was instructed to hit cross-court forehand drives as they would in a tennis match until three valid shots landed in the target box (Figure 1). The balls were thrown by a tennis ball machine (Lobster Elite model: 800.526.4041, North Hollywood, CA, USA) at a controlled velocity (24 m/s) as in other studies [2,4] with 3 s between each stroke [5], elevation 25 and spin 2.

After the 15 min warm-up, the participants performed eight isometric maximal voluntary contractions (IMVCs) of 3 s duration, repeated three times with a rest interval of 1 min, as previously described [15], for the following muscles (Table 1): anterior deltoid (AD), posterior deltoid (PD), biceps brachii (BB), triceps brachii (TB), flexor carpi radialis (FCR), extensor carpi radialis (ECR), pectoralis major (PM) and Latissimus dorsi (LD). This procedure allowed EMG activity, originally expressed in microvolts, to be normalized as a percentage of IMVC or a reference voluntary contraction [16]. This also allows the comparison of absolute values of muscular activity, thereby enabling comparisons between muscles or between subjects [17,18]. Then, each participant was instrumented with a full-body motion capture system suit using 17 inertial measurement unit (IMU) sensors (Xsens MVN Link System, Xsens technologies, Enschede, The Netherlands).

Afterwards, each participant performed repeated cross-court forehand drives (series of ten shots with a rest interval of 3 min) until three successful shots inside the target area were accomplished [5]. The impact zone was in the center of the court near the baseline, and the target box was defined in the right side of the opposite court, as a 3 × 4.5 m rectangle (Figure 1) [19]. All trials were conducted on an indoor clay tennis court.

**Table 1:** Standardized assessment protocol for determining the isometric maximal voluntary contraction (IMVC) of the eight muscles investigated in this study [15,20].

Muscle	Procedures for IMVC
Anterior deltoid (AD)	Arm internal rotation in the frontal plane at 90° shoulder flexion and 20° elbow flexion.
Posterior deltoid (PD)	Horizontal abduction in the sagittal plane at 90° shoulder abduction with forearm pronated.
Biceps brachii (BB)	The upper arm along the trunk, the forearm horizontal and supinated. Resistance applied downward against the wrist.
Triceps brachii (TB)	Sitting on a chair; the upper arm next to the trunk; the forearm horizontal and supinated. Resistance applied upward against the wrist.
Flexor carpi radialis (FCR)	Sitting on a chair with the forearm resting against the thigh and the fist supinated, downward resistance is applied to the hand.
Extensor carpi radialis (ECR)	Seated in a chair, with the forearm resting on the thigh and the fist pronated; upward resistance is applied to the hand.
Pectoralis major (PM)	Arm internal rotation in the frontal plane at 90° shoulder flexion and 20° elbow flexion.
Latissimus dorsi (LD)	The upper arm horizontal; the forearm vertical. Resistance applied frontward against the elbow.

### 2.3. Instrumentation

**Figure 2:**
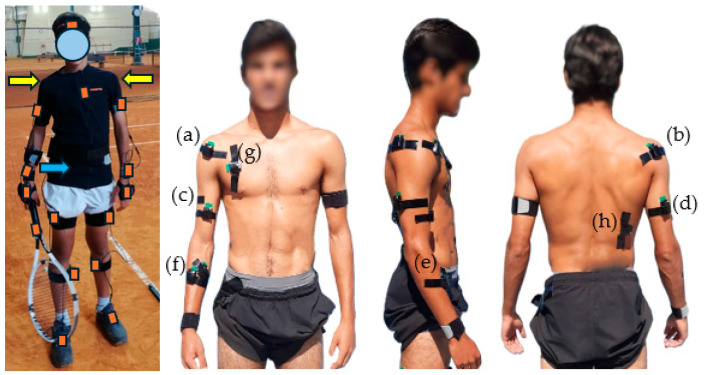
One participant with the 18 IMUs placed over the feet, shanks, thighs, pelvis (blue arrow), sternum, head, scapulae (yellow arrows), upper arms, forearms, hands and in the racket grip and the EMG sensor placement in one participant: (**a**) anterior deltoid, (**b**) posterior deltoid, (**c**) biceps brachii, (**d**) triceps brachii, (**e**) flexor carpi radialis, (**f**) extensor carpi radialis, (**g**) pectoralis major, (**h**) latissimus dorsi.

Kinematic and EMG data were synchronized in time between the IMU system and the Qualisys Trak Manager (version 2.10., Qualisys AB, Gothenburg, Sweden). The kinematic data were recorded at 240 Hz with a full-body IMUs suit using 17 IMU sensors (Xsens MVN Link System, Xsens technologies, Enschede, The Netherlands) and the Xsens MVN Analyse Pro software (version 2019.2) which were continuously updated using a biomechanical model of the human body [21]. The Xsens MVNV Link system consists of 17 IMUs (36 × 24 × 10 mm, 10 g), each containing 3D gyroscopes, 3D accelerometers and a magnetometer, connected to a body and battery pack. These sensors were placed over the feet, shanks, thighs, pelvis, sternum, head, scapulae, upper arms, forearms, hands and in the racket grip (Figure 2), according to the manufacturer’s instructions. All sensors, cables as well as the body and battery pack were incorporated inside a Lycra suit. Once all sensors were placed, the following anthropometric measures were taken from each participant to scale their avatar model: standing height, shoe length, arm span, ankle, knee, hip and shoulder heights, hip and shoulder widths, and shoe sole height [21,22]. To align the motion trackers to the segment, a calibration procedure was performed with the participants standing in an “n-pose” neutral position with the palm of the hands facing medially, “walking” and standing in “n-pose” again [21]. The IMUs operated with a biomechanical model incorporating six degrees of freedom (DOFs) joint laxity [22].

One Qualisys video camera (Figure 1, cam1), model 210c (Qualisys AB, Gothenburg, Sweden), operating at 240 Hz in Qualisys Track Manager software was synchronized with the IMUs to identify the acceleration phase (beginning of the forward swing until the instant of impact). One digital video camera (Panasonic HC-V10, Panasonic, Osaka, Japan) (Figure 1, cam 2) operating at 60 Hz captured the placement of the ball in the target zone.

The activity of eight selected muscles (AD, PD, BB, TB, FCR, ECR, PM and LD) of the dominant upper arm and trunk was recorded using surface electrodes (Trigno^TM^ Wireless System, Delsys, Inc., Boston, MA, USA; 2160 Hz; interelectrode distance = 1 cm; silver) using the Qualisys Trak Manager. The electrode placements followed the SENIAM recommendations to avoid EMG limitations [23]. The skin surface was shaved and cleaned with alcohol. EMG sensors were placed on the muscle belly aligned with the muscle fibers’ direction and fixed to the skin with double-sided tape and reinforced with tape around the sensor (Figure 2). A connection test was performed to verify if the EMG signal was reliable and whether the electrodes have been placed properly; these procedures were followed by the EMG normalization in amplitude.

### 2.4. Data Processing

Kinematic data were processed in high definition using MVN Analyse Pro software (version 2019.2) and exported in MVNX format for analysis in Visual3D (V2024.09.1, C-Motion, Inc., Germantown, MA, USA). A 15-segment biomechanical model (head, thorax, upper arms, forearms, hands, pelvis, thighs, shanks, and feet) was constructed. Segment position and orientation (POSE) data from the MVNX file were used to compute joint kinematics of the shoulder, elbow, wrist, hip, knee, and ankle, as well as the separation angle between the shoulders and pelvis in the transverse plane. Joint angles for the dominant side (rear leg) were computed using a medio-lateral, antero-posterior, axial Cardan sequence rotation [24].

EMG raw signals were filtered (Butterworth order 1, band-pass 10 Hz and 500 Hz). Before computing the root mean square (RMS) values (EMGrms, 50 ms), the maximum EMGrms values were obtained from the EMG signals recorded during the IMVC, and the mean of the peak values across the three repetitions was used as the normalization reference. Additionally, the mean EMGrms values were calculated from the EMG signals collected during the acceleration phase of the forehand drive, which was defined as the period from the start of the racket movement in the direction of the ball to impact. Finally, the average normalized EMG values of both groups (HP and INT) were expressed as a percentage of the EMG level measured during the IMVC [5]. Both kinematic and EMG data were time-normalized (0–100%) from the beginning of the acceleration phase until ball impact (forward swing).

For each participant, kinematic and EMG data from three successful cross-court forehand drives were selected and analyzed from the beginning of the acceleration phase until ball impact (forward swing). Joint angle and joint angular velocity time series were computed for the dominant shoulder (abduction/adduction, flexion/extension, internal/external rotation), elbow (flexion/extension, forearm pronation/supination), wrist (flexion/extension, radial/ulnar deviation), trunk–pelvis separation angle, hip (abduction/adduction, flexion/extension, internal/external rotation), knee (flexion/extension) and ankle (dorsiflexion/plantarflexion), and linear velocities were calculated for the hip, shoulder, elbow, wrist and racket head. In the same time window, EMG amplitude waveforms were obtained from eight muscles of the dominant upper limb and trunk: anterior deltoid, posterior deltoid, pectoralis major, latissimus dorsi, biceps brachii, triceps brachii, flexor carpi radialis and extensor carpi radialis.

### 2.5. Statistical Analyses

Baseline comparisons were performed to verify sample homogeneity. Age was not normally distributed in the HP group and was therefore compared using the Mann–Whitney U test, whereas height, body mass, and BMI were compared using independent-samples *t*-tests. A priori power analysis of statistical parametric mapping (SPM) two-sample *t*-test was conducted using Power1D toolbox [25,26]. The parameters required for the sample size calculation were estimated based on the results of a previous study [4] on maximum shoulder linear velocity (which was the only relevant variable with significant between-group effect) of tennis forehand drive of the elite and high-performance players [27]. The result revealed that a minimum of seven participants was required in each group to achieve a statistical power of 0.8.

To examine the effect of expertise on these waveforms, multiple one-dimensional statistical parametric mapping (SPM1D) two-tailed two-sample *t*-tests (spm1d.stat.ttest2) were performed using the open-source spm1d Python package (version: v0.4; https://spm1d.org; accessed on 11 March 2026) [28,29]. The SPM-1D method uses random field theory to identify field regions that co-vary significantly with the experimental design [30,31,32]. The significance level was set at *p* < 0.05, and Bonferroni-corrected thresholds were applied to control Type I errors associated with multiple comparisons of the kinematic (0.05 ÷ 29 ≈ 0.0017) and EMG (0.05 ÷ 8 ≈ 0.0063) variables. Effect sizes were quantified (Appendix A) using Cohen’s *d*, with thresholds of 0.20, 0.50, and 0.80 indicating small, medium, and large effects, respectively [33].

Normality of each continuous waveform was assessed using the built-in SPM normality test (spm1d.stats.normality.ttest2). Shoulder, elbow, and wrist linear velocities; joint angles including shoulder flexion/extension and internal/external rotation, elbow pronation/supination, wrist flexion/extension and radial/ulnar deviation, trunk–pelvis separation, and hip abduction/adduction; as well as all analyzed angular velocity and EMG variables did not meet the assumption of normality. Accordingly, these variables were analyzed using the nonparametric SPM two-sample *t*-test (spm1d.stats.nonparam.ttest2).

## 3. Results

**Figure 3:**
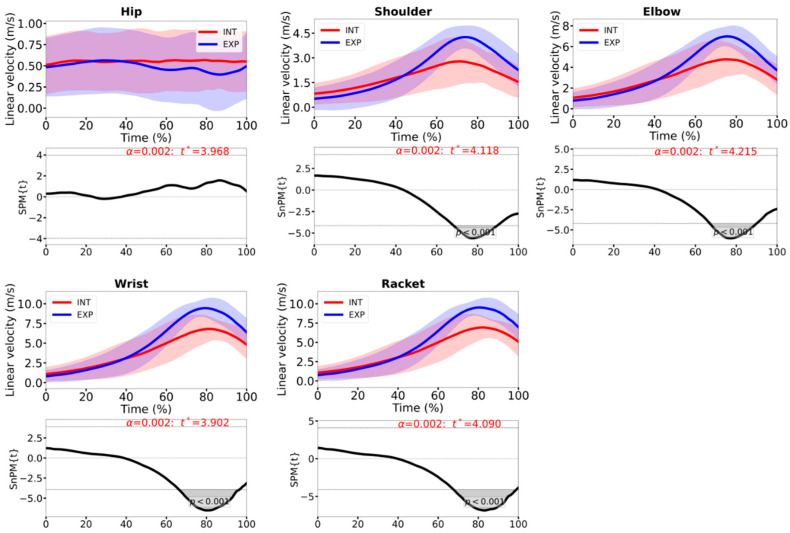
Linear velocities and the respective 1D-SPM analysis, during the time-normalized forward swing, for the INT (red line) and the HP tennis players (blue line). Hip, shoulder, elbow, wrist and racket linear velocity. The dashed line indicates the critical threshold t*. Grey shaded regions where SPM{t}/SnPM{t} exceeds t* are statistically significant.

No significant between-group differences were found for age (*p* = 0.4), height (*p* = 0.3), body mass (*p* = 0.1) or BMI (*p* = 0.3), supporting the comparability of the two groups in terms of baseline anthropometric characteristics. The linear racket velocity was significantly greater in the HP group during the end of the forward swing, and this was accompanied by higher shoulder, elbow and wrist linear velocities (all *p* < 0.001; Figure 3). Hip linear velocity did not differ between groups.

**Figure 4:**
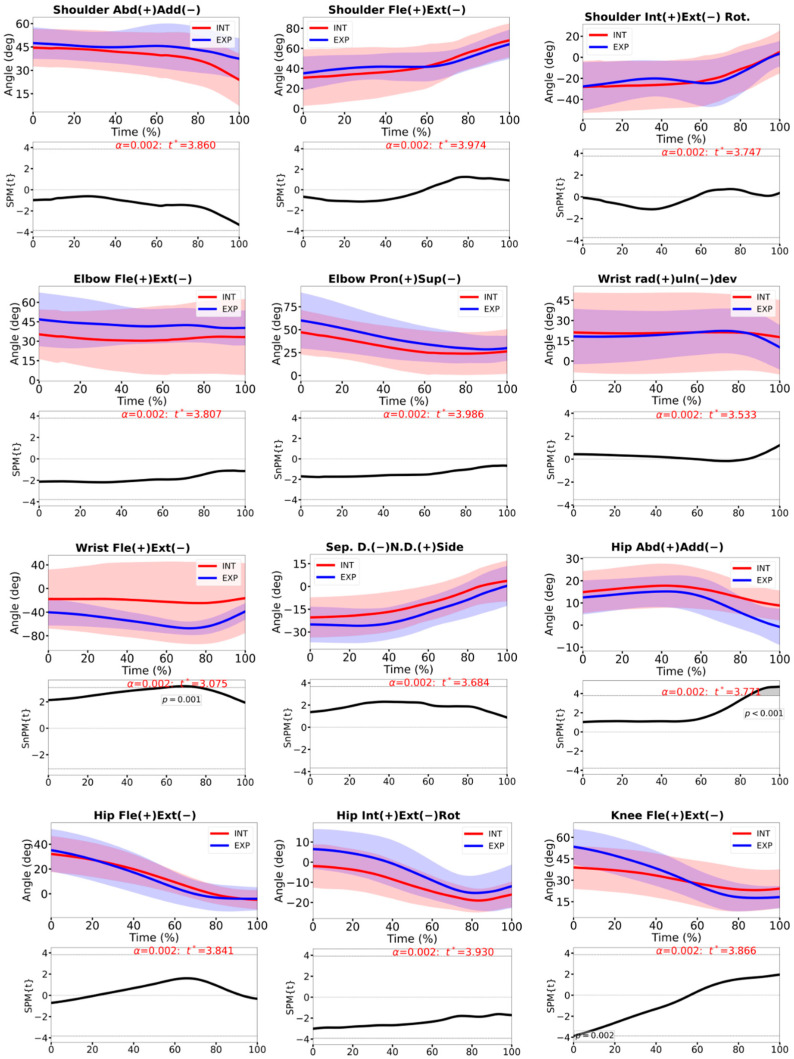
Joint angles and the respective 1D-SPM analysis, during the time-normalized forward swing, for INT (red line) and the HP players. Shoulder abduction/adduction, shoulder flexion/extension, shoulder internal/external rotation, elbow flexion/extension, pronation/supination, hand radial/ulnar deviation, wrist flexion/extension, separation angle, hip abduction/adduction, hip flexion/extension, hip internal/external rotation, knee flexion. The dashed line indicates the critical threshold t*. Grey shaded regions where SPM{t}/SnPM{t} exceeds t* are statistically significant.

Overall joint angle patterns were broadly similar between groups throughout the stroke (Figure 4). The statistical parametric mapping revealed only a few localized differences where HP players performed the forehand with a more extended wrist across most of the forward swing (*p* < 0.001), slightly greater early knee flexion (*p* = 0.002), whereas INT players maintained greater hip abduction near the end of the forward swing (*p* < 0.001). No other consistent differences in upper- or lower-limb joint angles were detected.

**Figure 5:**
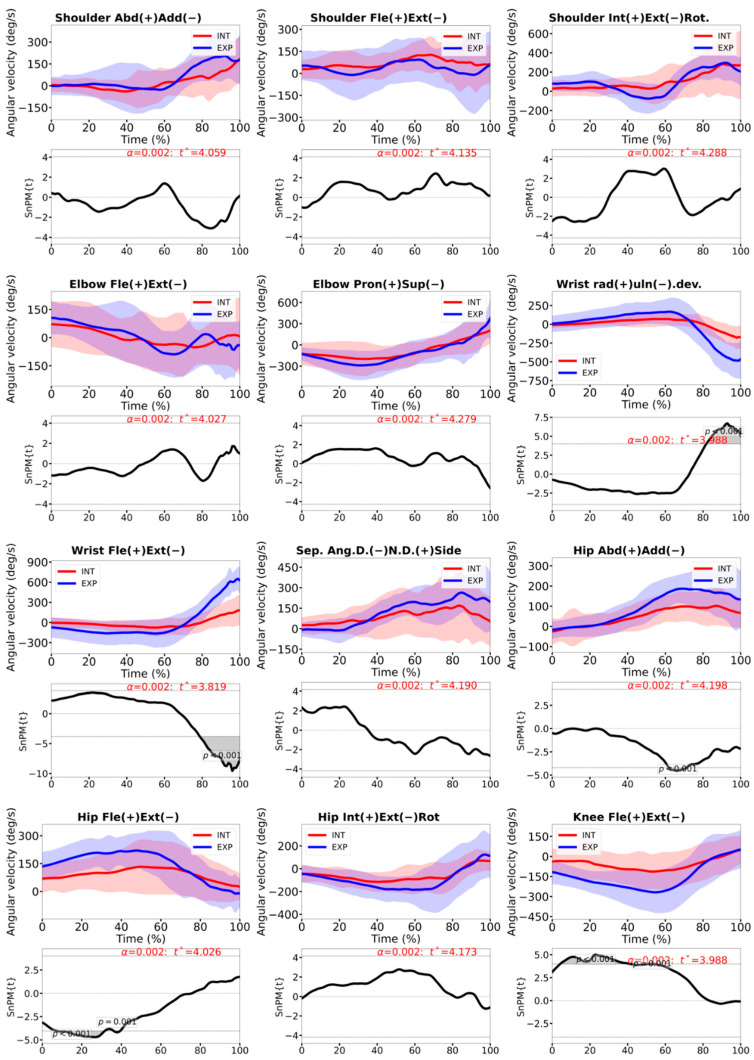
Joint angular velocities and the respective 1D-SPM analysis, during the time-normalized forward swing, for INT (red line) and the HP players. Shoulder abduction/adduction, shoulder flexion/extension, shoulder internal/external rotation, elbow flexion/extension, pronation/supination, hand radial/ulnar deviation, wrist flexion/extension, separation angle, hip flexion/extension, knee flexion. The dashed line indicates the critical threshold t*. Grey shaded regions where SPM{t}/SnPM{t} exceeds t* are statistically significant.

Angular velocity profiles showed group differences mainly in the lower limbs and distal upper limb (Figure 5). HP players exhibited higher hip and knee flexion/extension angular velocities during the first half of the forward swing (both *p* < 0.001) and greater wrist angular velocities in both flexion/extension and radial/ulnar deviation close to impact (*p* < 0.001). Shoulder and elbow angular velocities and trunk–pelvis separation angular velocity did not differ significantly between groups.

**Figure 6:**
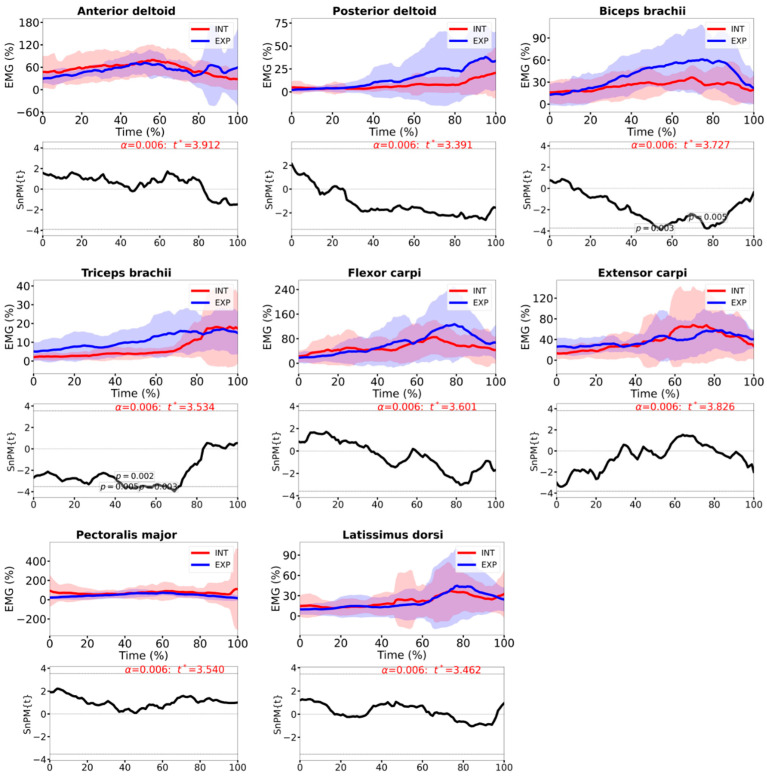
EMG amplitude activity during the time-normalized forward swing in the selected muscles. Anterior deltoid, posterior deltoid, biceps brachii, triceps brachii, flexor carpi radialis, extensor carpi radialis, pectoralis major and latissimus dorsi. The dashed line indicates the critical threshold t*. Grey shaded regions where SPM{t}/SnPM{t} exceeds t* are statistically significant.

The normalized EMG patterns were comparable between HP and INT players (Figure 6) with increasing activity during the forward swing and peaking close to impact. The SPM analysis identified significant group differences in the BB EMG with greater amplitude in the HP players than in the INT group from approximately 45 to 80% of the forward swing (*p* = 0.003–0.005). TB activity was also higher in the HP group over a shorter interval in the second half of the swing (around 55 to 75%; *p* = 0.002). For the remaining muscles, AD, PD, FCR, ECR, PM and LD, the SPM trajectories did not exceed the critical threshold, indicating no statistically significant between-group differences across the swing (*p* > 0.01).

## 4. Discussion

The main purpose of this study was to compare the kinematic and neuromechanical characteristics of the tennis forehand drive between HP and INT tennis players, with a particular focus on upper-limb and trunk EMG amplitude. The main findings were that HP players generated clearly greater racket and distal segment linear velocities, showed only small and temporally localized differences in joint angles and angular velocities, and displayed broadly similar time-varying EMG amplitude profiles in most upper-limb and trunk muscles. However, the elbow flexor/extensor pair differed between groups, with HP players displaying higher BB activation in two significant clusters between approximately 45 and 80% of the forward swing (*p* = 0.003–0.006) and greater TB activation during the late forward swing (*p* < 0.01). Thus, the hypothesis that advanced players would achieve higher racket velocity was supported, while the expectation of large, systematic differences in EMG activation profiles across all muscles was only partially confirmed.

The higher racket and distal segment velocities observed in the HP group (Figure 3) are in line with previous kinematic studies showing that higher level players exhibit greater racket-head speed and more efficient segmental motion during the forehand [1,2,4]. In our data, these differences were particularly evident during the final part of the forward swing, just before the impact where HP players displayed higher linear velocities at the wrist, elbow and shoulder. This pattern is consistent with the concept of a well-organized kinetic chain, in which energy is generated and transferred from the lower limbs and trunk to the upper extremity and racket [3,34]. Recent IMU-based work has shown that shoulder horizontal flexion, elbow extension and shoulder internal rotation are major contributors to racket-head speed in attacking forehands [19], and our observation of higher distal linear velocities in HP players fits well within this framework of coordinated proximal-to-distal sequencing.

At the kinematic level (Figure 4), the overall similarity of joint angle trajectories between groups suggests that both HP and INT players use broadly comparable movement patterns for the forehand drive and are consistent with previous studies [1]. The differences that did emerge included a more extended wrist throughout the forward swing and greater early knee flexion in HP players and higher hip and knee angular velocities in the HP group. A more extended wrist has been associated with effective forehand technique and an advantageous alignment of the racket–forearm complex for both power and control [3]. The greater early knee flexion and faster lower-limb extension in HP players suggest a deeper loading position at the start of the acceleration phase and a more dynamic use of the legs to initiate the stroke, contributing to the forward and upward drive of the body and racket. However, because no EMG data were collected from the lower-limb muscles, these kinematic differences cannot be directly interpreted in neuromuscular terms. The initial knee position has been positively associated with a higher racket velocity [35]. In contrast, the greater late hip abduction in the INT players may reflect less efficient weight transfer or a tendency to “open” the pelvis without fully coupling it to trunk rotation, which might reduce the effectiveness of energy transfer along the chain [4,34].

Near impact, the HP also showed higher wrist angular velocities (Figure 5) in both flexion/extension and radial/ulnar deviation. The greater wrist angular velocity observed in the HP players is interpreted here primarily as a distal expression of more effective proximal-to-distal sequencing, rather than as evidence that the wrist acts as an isolated active driver of the stroke. This interpretation is consistent with previous biomechanical evidence showing that the contribution of the hand/wrist segment to racket-head velocity is comparatively small (4.5–5.3% for flexion/extension and 4.9–6.1% for adduction/abduction) [19], whereas the upper arm and forearm make substantially larger contributions. Therefore, the wrist’s “whip-like” behavior may reflect the transmission and release of momentum generated more proximally along the kinetic chain, although some active contribution from the distal musculature cannot be excluded. Taken together, these findings suggest that, relative to INT players, HP athletes do not adopt radically different joint postures, but rather generate and transmit force more effectively along the kinetic chain—from the legs and pelvis through the trunk to the distal segments—culminating in a faster “whipping” action of the wrist. This interpretation is consistent with previous descriptions of efficient proximal-to-distal sequencing in high-level forehands, where lower-limb drive and trunk rotation are key contributors to racket-head speed [3,4,34].

Our initial hypothesis of level-dependent differences in upper-limb muscle activity was only partially supported. SPM revealed significant between-group differences in time-varying EMG amplitude for the elbow flexors and extensors: HP players showed greater biceps brachii activation during two intervals in the mid-to-late forward swing (≈45–80% of the movement; *p* = 0.003–0.006) and higher triceps brachii activation in the final part of the swing (≈70–90%; *p* < 0.001). Contrary to Goislard de Monsabert et al. [10], we did not observe level-dependent differences in FCR or ECR activation, as the other studied muscles. In both groups, EMG activity increased towards impact, with peaks occurring during racket acceleration and ball contact, consistent with earlier descriptions of forehand muscle recruitment in the shoulder and arm [8]. The very high relative activation levels reported for FCR and ECR during powerful forehands [9] were qualitatively evident in our data in both HP and INT players, underlining the substantial demands on the wrist flexor–extensor complex, but these demands did not differ statistically between levels.

Taken together, these results suggest that HP players do not achieve higher racket velocities by simply increasing muscle activation compared to intermediate players. Instead, they appear to attain superior stroke performance while using similar levels of muscular effort, by organizing joint motion and segmental timing more effectively, particularly in the lower limbs and distal wrist segment. From a motor control perspective [36], this may reflect a more effective organization of the available degrees of freedom and a more refined proximal-to-distal sequencing pattern in the HP players. This interpretation is consistent with broader biomechanical and motor control models of stroke production, which emphasize that performance in tennis depends primarily on efficient kinetic chain coordination and segmental sequencing, rather than on isolated increases in muscle output [3,34]. It may also help reconcile our findings with those of Goislard de Monsabert et al. [10], who reported higher FCR activation in intermediate players and greater ECR activation in advanced players: differences in task constraints (e.g., topspin versus flat strokes, ball speed or tactical context) and grip strategies may shift the balance of flexor–extensor demand, whereas in the present protocol both groups may have converged on similar neuromuscular solutions.

From an applied perspective, the combination of higher racket speed and similar EMG amplitude in advanced players has important implications for both performance and injury risk. Epidemiological data indicate a high prevalence of upper-limb overuse injuries in tennis, particularly at the wrist and elbow [37], with ulnar-sided wrist pathologies such as extensor carpi ulnaris tendinopathy frequently reported in competitive players [11]. The fact that both groups in our study displayed high relative EMG amplitudes in forearm muscles suggests that the wrist and forearm are consistently heavily loaded, independent of playing level. However, if HP players can generate greater racket speed without proportionally higher EMG, this implies a more favorable ratio between performance and neuromuscular load. Conversely, INT players who attempt to increase ball speed primarily through the distal segments, rather than through improved whole-body coordination, may be at greater risk of overloading the wrist and forearm. Coaches and clinicians might therefore prioritize technical interventions that enhance lower-limb contribution and proximal-to-distal sequencing, rather than focusing solely on local strengthening of the upper limb.

Methodologically, this study adds to the growing literature using one-dimensional statistical parametric mapping to examine continuous kinematic and EMG signals in tennis strokes. By analyzing the entire time-normalized forehand cycle, rather than a small number of discrete events, SPM allows the detection of temporally localized differences that might otherwise be missed. Although the present data were obtained with an IMU-based system, a recent work has demonstrated good concurrent validity of IMU-based systems for capturing forehand kinematics and reproducing SPM results in comparison with laboratory-based motion capture [38]. Moreover, a key strength of this study is that it was conducted on court, with players performing forehand drives under realistic playing conditions, which enhances its ecological validity and practical relevance.

Some limitations should be acknowledged. Although a priori power analysis was performed, it was based on shoulder linear velocity because this was the only variable with previously reported between-group differences in a comparable tennis forehand task. In contrast, comparable EMG waveform data comparing players of different skill levels are scarce, which prevented a reliable a priori power estimation for EMG outcomes. Therefore, given the modest sample size and the greater inter-individual variability typically observed in EMG measures, the possibility of Type II errors should be considered in the EMG comparisons. In particular, the absence of significant differences in muscles such as FCR and ECR may reflect either true similarity between groups or insufficient statistical power to detect subtle effects. In addition, only eight upper-limb and trunk muscles were recorded. Therefore, potential differences in key lower-limb muscles (e.g., rectus femoris and gluteus maximus) involved in force generation and transfer were not assessed, limiting a more direct neuromuscular interpretation of the kinetic chain. Likewise, trunk EMG assessment was limited to the pectoralis major and latissimus dorsi, which are relevant for upper-body motion but are not the primary muscles responsible for trunk rotation. The absence of EMG data from key core muscles, such as the internal and external obliques and erector spinae, limits a more comprehensive understanding of segmental coordination during the forehand drive. At present, no consensus exists on which EMG normalization methods reliably elicit maximal activation, as their effectiveness is both muscle- and task-dependent [18,39]. To our knowledge, comparison of different EMG normalization methods for upper extremities during tennis forehand has never been conducted before. EMG normalization to isometric IMVC, while standard, may not perfectly reflect maximal activation capacity during dynamic stretch–shortening actions [39], such as the forehand drive [9]. As a result, similar normalized EMG amplitudes between HP and INT players may mask differences in relative neuromuscular effort or efficiency. For example, HP players may achieve higher racket speeds while operating at a lower percentage of their task-specific activation capacity, whereas INT players may rely on a higher relative level of muscle activation to achieve lower performance outcomes. Alternative normalization approaches using dynamic reference contractions could therefore alter the scaling of EMG amplitudes and shift interpretation toward differences in neuromuscular demand rather than absolute activation magnitude. However, this approach should be employed only when the researcher is confident that the task designed to elicit the dynamic MVC engages all muscles under investigation uniformly and, ideally, to their maximal capacity [18]. In addition, performance-level differences may also be reflected in the temporal features of muscle activation, such as a faster rate of EMG rise, rather than in amplitude alone. Such differences would not be fully captured by either IMVC-based or dynamic normalization approaches, as both primarily affect amplitude scaling rather than activation timing. Future studies should therefore systematically evaluate and compare different EMG normalization methods for upper-extremity muscles during the tennis forehand to better capture skill-level differences in neuromuscular demand. Finally, the study focused on a single type of forehand under relatively controlled conditions; different spin types, ball speeds or tactical situations may elicit distinct kinematic and EMG strategies.

In summary, the aim of this study was to compare kinematics and upper-limb/trunk EMG amplitude between HP and INT tennis players during the forehand drive. The results showed that the main between-group differences were kinematic, with HP players producing higher racket and distal segment velocities, whereas EMG amplitude in the analyzed upper-limb and trunk muscles was largely similar between groups. These findings indicate that superior forehand performance in HP players is associated primarily with more effective segmental coordination and kinetic chain timing rather than with greater overall muscle activation. Future research should extend this approach to a wider range of stroke types and competitive levels, integrate a broader set of muscles, especially in the lower limbs, and explore how technical training can best optimize both racket speed and forearm load in line with the current knowledge of overuse injury mechanisms in tennis.

## Figures and Tables

**Figure A1:**
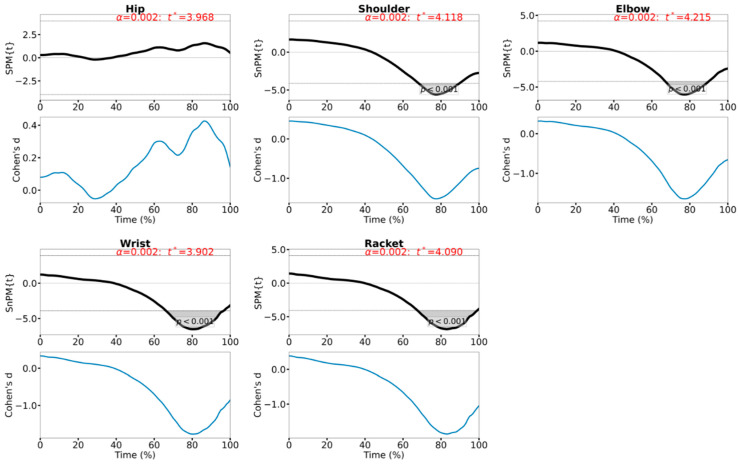
One-dimensional SPM{t}/SnPM{t} results (**upper panels**) and corresponding time-varying effect sizes (Cohen’s *d*; **lower panels**) for the between-group comparison of linear velocity waveforms of the hip, shoulder, elbow, wrist, and racket during the forward swing of the tennis forehand drive. Red text indicates the Bonferroni-corrected significance level and critical threshold. Significant supra-threshold clusters are labeled with their corresponding *p*-values. Positive Cohen’s *d* values indicate greater values in the HP group, whereas negative values indicate greater values in the INT group. The dashed line indicates the critical threshold t*. Grey shaded regions where SPM{t}/SnPM{t} exceeds t* are statistically significant.

**Figure A2:**
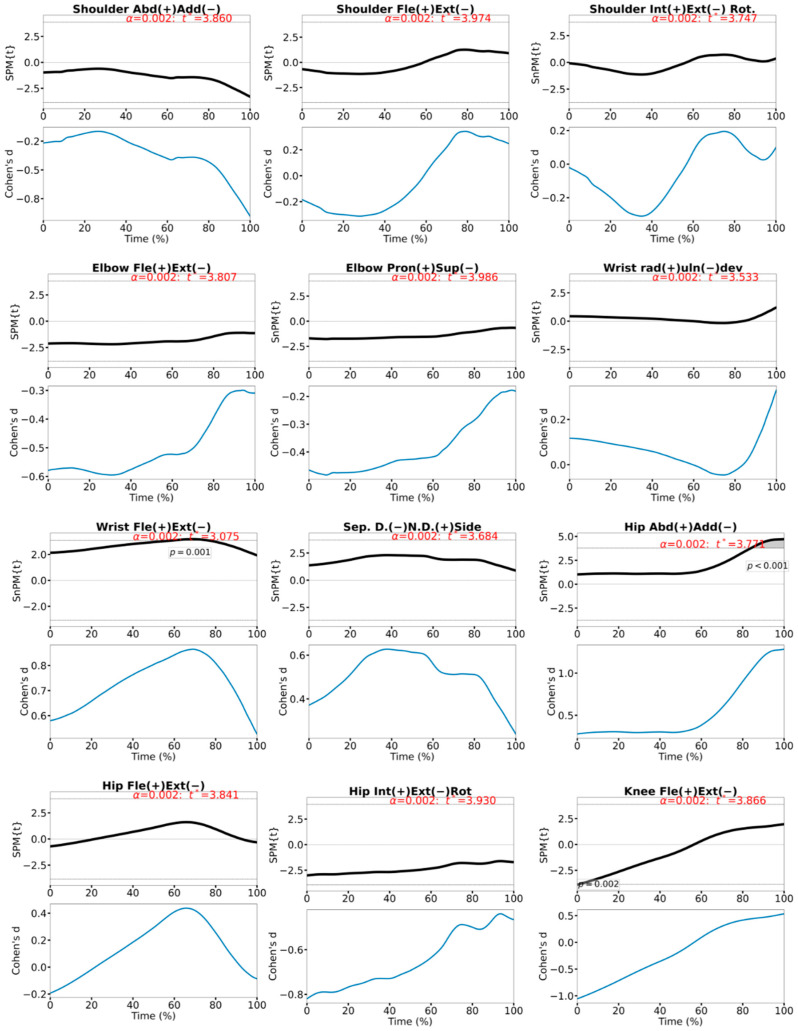
One-dimensional SPM{t}/SnPM{t} results (**upper panels**) and corresponding time-varying effect sizes (Cohen’s *d*; **lower panels**) for the between-group comparison of joint-angle waveforms during the forward swing of the tennis forehand drive. Red text indicates the Bonferroni-corrected significance level and critical threshold. Significant supra-threshold clusters, when present, are labeled with their corresponding *p*-values. Positive Cohen’s *d* values indicate greater values in the HP group, whereas negative values indicate greater values in the INT group. The dashed line indicates the critical threshold t*. Grey shaded regions where SPM{t}/SnPM{t} exceeds t* are statistically significant.

**Figure A3:**
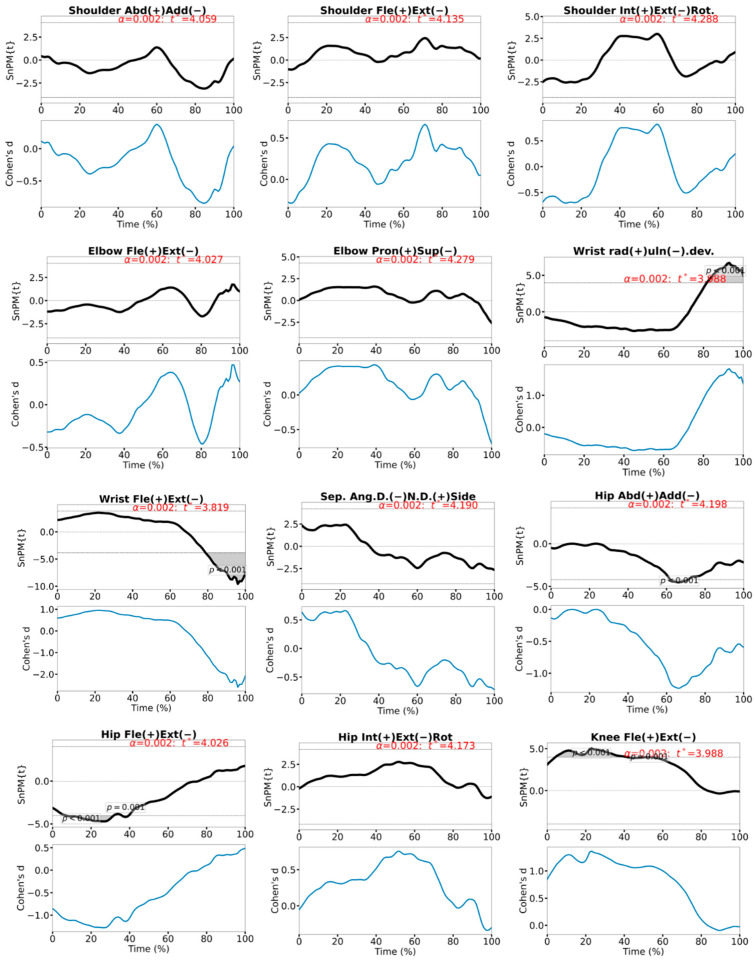
One-dimensional SPM{t}/SnPM{t} results (**upper panels**) and corresponding time-varying effect sizes (Cohen’s *d*; **lower panels**) for the between-group comparison of joint-angular velocities waveforms during the forward swing of the tennis forehand drive. Red text indicates the Bonferroni-corrected significance level and critical threshold. Significant supra-threshold clusters are labeled with their corresponding *p*-values. Positive Cohen’s *d* values indicate greater values in the HP group, whereas negative values indicate greater values in the INT group. The dashed line indicates the critical threshold t*. Grey shaded regions where SPM{t}/SnPM{t} exceeds t* are statistically significant.

**Figure A4:**
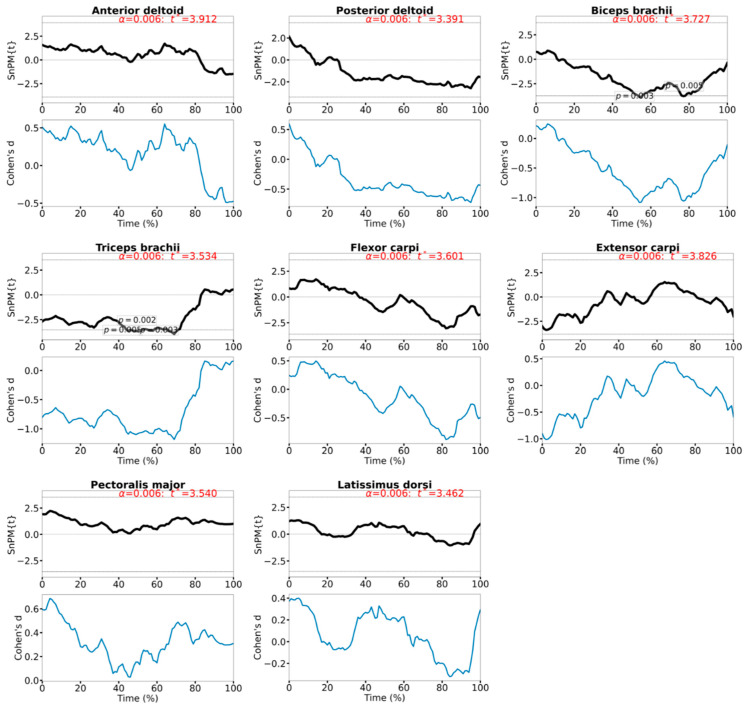
One-dimensional SPM{t}/SnPM{t} results (**upper panels**) and corresponding time-varying effect sizes (Cohen’s *d*; **lower panels**) for the between-group comparison of normalized EMG amplitude waveforms of the analyzed upper-limb and trunk muscles during the forward swing of the tennis forehand drive. Red text indicates the Bonferroni-corrected significance level and critical threshold. Significant supra-threshold clusters are labeled with their corresponding *p*-values. Positive Cohen’s *d* values indicate greater values in the HP group, whereas negative values indicate greater values in the INT group. The dashed line indicates the critical threshold t*. Grey shaded regions where SPM{t}/SnPM{t} exceeds t* are statistically significant.

## Data Availability

The data presented in this study is available on request from the corresponding author.

## Abbreviations

The following abbreviations are used in this manuscript:

EMG	Electromyography
MVC	Maximal voluntary contraction
AD	Anterior deltoid
PD	Posterior deltoid
BB	Biceps brachii
TB	Triceps brachii
FCR	Flexor carpi radialis
ECR	Extensor carpi radialis
PM	Pectoralis major
LD	Latissimus dorsi
IMU	Inertial measurement unit
DOF	Degrees of freedom
RMS	root mean square
HP	High-performance
INT	Intermediate
SPM-1D	One-dimensional statistical parametric mapping

## References

[1] Seeley M.K., Funk M.D., Denning W.M., Hager R.L., Hopkins J.T. (2011). Tennis forehand kinematics change as post-impact ball speed is altered. Sports Biomech..

[2] Landlinger J., Lindinger S.J., Stöggl T., Wagner H., Müller E. (2010). Kinematic differences of elite and high-performance tennis players in the cross court and down the line forehand. Sports Biomech./Int. Soc. Biomech. Sports.

[3] Roetert E.P., Kovacs M., Knudson D., Groppel J.L. (2009). Biomechanics of the Tennis Groundstrokes: Implications for Strength Training. Strength. Cond. J..

[4] Landlinger J., Lindinger S., Stöggl T., Wagner H., Müller E. (2010). Key factors and timing patterns in the tennis forehand of different skill levels. J. Sports Sci. Med..

[5] Rogowski I., Rouffet D., Lambalot F., Brosseau O., Hautier C. (2011). Trunk and upper limb muscle activation during flat and topspin forehand drives in young tennis players. J. Appl. Biomech..

[6] Rogowski I., Creveaux T., Faucon A., Rota S., Champely S., Guillot A., Hautier C. (2009). Relationship between muscle coordination and racket mass during forehand drive in tennis. Eur. J. Appl. Physiol..

[7] Rota S., Morel B., Saboul D., Rogowski I., Hautier C. (2014). Influence of fatigue on upper limb muscle activity and performance in tennis. J. Electromyogr. Kinesiol..

[8] Van Gheluwe B., Hebbelinck M. (1986). Muscle actions and ground reaction forces in tennis. Int. J. Sport Biomech..

[9] Loushin S.R., Kakar S., Tetzloff S.U., Lubbers P., Ellenbecker T.S., Kaufman K.R. (2022). Upper Extremity Kinematics and Electromyographic Activity in Uninjured Tennis Players. Appl. Sci..

[10] Goislard de Monsabert B., Herbaut A., Cartier T., Vigouroux L. (2023). Electromyography-informed musculoskeletal modeling provides new insight into hand tendon forces during tennis forehand. Scand. J. Med. Sci. Sports.

[11] Montalvan B., Parier J., Brasseur J.L., Le Viet D., Drape J.L. (2006). Extensor carpi ulnaris injuries in tennis players: A study of 28 cases. Br. J. Sports Med..

[12] Kinel E., D’Amico M., Roncoletta P. (2018). Normative 3D opto-electronic stereo-photogrammetric sagittal alignment parameters in a young healthy adult population. PLoS ONE.

[13] De Blasiis P., Fullin A., Sansone M., Perna A., Caravelli S., Mosca M., De Luca A., Lucariello A. (2022). Kinematic Evaluation of the Sagittal Posture during Walking in Healthy Subjects by 3D Motion Analysis Using DB-Total Protocol. J. Funct. Morphol. Kinesiol..

[14] Camuncoli F., Barni L., Nutarelli S., Rocchi J.E., Barcillesi M., Di Dio I., Sambruni A., Galli M. (2022). Validity of the Baiobit Inertial Measurements Unit for the Assessment of Vertical Double- and Single-Leg Countermovement Jumps in Athletes. Int. J. Environ. Res. Public Health.

[15] Chow J.W., Carlton L.G., Lim Y.T., Shim J.H., Chae W.S., Kuenster A.F. (1999). Muscle activation during the tennis volley. Med. Sci. Sports Exerc..

[16] Lehman G.J., Mcgill S.M. (1999). The Importance of Normalization in the Interpretation of Surface Electromyography. J. Manip. Physiol. Ther..

[17] Soderberg G.L., Knutson L.M. (2000). A Guide for Use and Interpretation of Kinesiologic Electromyographic Data. Phys. Ther..

[18] Burden A. (2010). How should we normalize electromyograms obtained from healthy participants? What we have learned from over 25 years of research. J. Electromyogr. Kinesiol..

[19] Pedro B., João F., Lara J.P.R., Cabral S., Carvalho J., Veloso A.P. (2022). Evaluation of Upper Limb Joint Contribution to Racket Head Speed in Elite Tennis Players Using IMU Sensors: Comparison between the Cross-Court and Inside-Out Attacking Forehand Drive. Sensors.

[20] Rota S., Rogowski I., Champely S., Hautier C. (2013). Reliability of EMG normalisation methods for upper-limb muscles. J. Sports Sci..

[21] Roetenberg D., Luinge H., Slycke P. (2013). Xsens MVN: Full 6dof human motion tracking using miniature inertial sensors. Xsens Technol..

[22] Myn U., Link M., Awinda M. (2018). Xsens MVN User Manual.

[23] Hermens H.J., Freriks B., Disselhorst-Klug C., Rau G. (2000). Development of recommendations for SEMG sensors and sensor placement procedures. J. Electromyogr. Kinesiol..

[24] Grood E.S., Suntay W.J. (1983). A Joint Coordinate System for the Clinical Description of Three-Dimensional Motions: Application to the Knee. J. Biomech. Eng..

[25] Robinson M.A., Vanrenterghem J., Pataky T.C. (2021). Sample size estimation for biomechanical waveforms: Current practice, recommendations and a comparison to discrete power analysis. J. Biomech..

[26] Pataky T.C. (2017). Power1D: A Python toolbox for numerical power estimates in experiments involving one-dimensional continua. PeerJ Comput. Sci..

[27] Luciano F., Ruggiero L., Pavei G. (2021). Sample size estimation in locomotion kinematics and electromyography for statistical parametric mapping. J. Biomech..

[28] Pataky T.C. (2010). Generalized n-dimensional biomechanical field analysis using statistical parametric mapping. J. Biomech..

[29] Pataky T.C. (2012). One-dimensional statistical parametric mapping in Python. Comput. Methods Biomech. Biomed. Eng..

[30] Pataky T.C., Robinson M.A., Vanrenterghem J. (2016). Region-of-interest analyses of one-dimensional biomechanical trajectories: Bridging 0D and 1D theory, augmenting statistical power. PeerJ.

[31] Pataky T.C., Robinson M.A., Vanrenterghem J. (2013). Vector field statistical analysis of kinematic and force trajectories. J. Biomech..

[32] Friston K.J., Holmes A.P., Worsley K.J., Poline J.P., Frith C.D., Frackowiak R.S. (1995). Statistical parametric maps in functional imaging: A general linear approach. Human Brain Mapp..

[33] Cohen J. (2013). Statistical Power Analysis for the Behavioral Sciences.

[34] Elliott B. (2006). Biomechanics and tennis. Br. J. Sports Med..

[35] Nesbit S.M., Serrano M., Elzinga M. (2008). The role of knee positioning and range-of-motion on the closed-stance forehand tennis swing. J. Sports Sci. Med..

[36] Latash M.L., Levin M.F., Scholz J.P., Schöner G. (2010). Motor Control Theories and Their Applications. Medicina.

[37] Kibler W.B., Safran M. (2005). Tennis Injuries. Med. Sport Sci..

[38] Pedro B., Cabral S., Veloso A.P. (2021). Concurrent validity of an inertial measurement system in tennis forehand drive. J. Biomech..

[39] Ball N., Scurr J. (2013). Electromyography Normalization Methods for High-Velocity Muscle Actions: Review and Recommendations. J. Appl. Biomech..

